# Alternative autophagy: mechanisms and roles in different diseases

**DOI:** 10.1186/s12964-022-00851-1

**Published:** 2022-03-31

**Authors:** Hong Feng, Nian Wang, Nan Zhang, Hai-han Liao

**Affiliations:** 1 Department of Geriatrics, Renmin Hospital of Wuhan University, Wuhan, 430060 Hubei People’s Republic of China; 2 Department of Cardiology, Renmin Hospital of Wuhan University, Wuhan, 430060 People’s Republic of China; 3 Hubei Key Laboratory of Metabolic and Chronic Diseases, Wuhan, 430060 People’s Republic of China

**Keywords:** Alternative autophagy, Canonical autophagy, Non-canonical autophagy, Mechanism, Diseases

## Abstract

As an important mechanism to maintain cellular homeostasis, autophagy exerts critical functions via degrading misfolded proteins and damaged organelles. Recent years, alternative autophagy, a new type of autophagy has been revealed, which shares similar morphology with canonical autophagy but is independent of Atg5/Atg7. Investigations on different diseases showed the pivotal role of alternative autophagy during their physio-pathological processes, including heart diseases, neurodegenerative diseases, oncogenesis, inflammatory bowel disease (IBD), and bacterial infection. However, the studies are limited and the precise roles and mechanisms of alternative autophagy are far from clear. It is necessary to review current research on alternative autophagy and get some hint in order to provide new insight for further study.

**Video Abstract**

**Supplementary Information:**

The online version contains supplementary material available at 10.1186/s12964-022-00851-1.

## Background

During physiological and pathological process, proteins and organelles are degraded continuously for maintaining normal function and protecting against unfavorable stress. Autophagy is one of the vital cellular mechanisms exerting such functions and it could be induced by many stressors, including but not limited to starvation, DNA damage, oxidative stress, heart shock [1, 2]. When the regulating function of autophagy is abnormal, misfolded proteins and damaged organelles are accumulated and the cellular homeostasis is disrupted. The deficiency of autophagy is closely related to various diseases, such as cardiovascular diseases [3], neurodegenerative diseases [4], oncogenesis [5], inflammatory bowel diseases [6], and etc.

Three types of autophagy have been revealed during the past years, including macro-autophagy, micro-autophagy and chaperone-mediated autophagy. As the main pathway to degrade superfluous proteins and damaged organelles, macro-autophagy is featured with the formation of isolation membranes, autophagosomes and autolysosomes during the process [7]. The term “autophagy”is generally used as the synonym with macro-autophagy. Isolation membranes are double membrane structure generating after the initiation of autophagy, which later expand and enclose intracellular constituents to mature into autophagosomes. Autophagosomes fuse with lysosomes to become autolysosomes and finally digest the engulfed cargos.

## Canonical autophagy and alternative autophagy

**Fig. 1:**
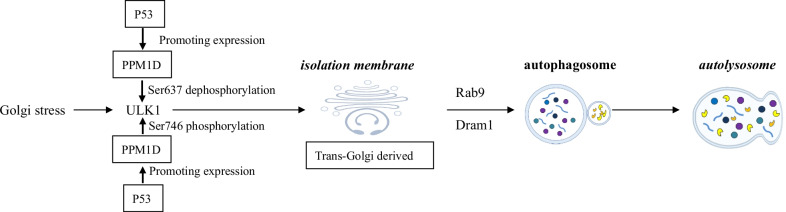
The molecular mechanism of alternative autophagy. Upon stimulation of Golgi stress, Ulk1 is dephosphorylated at Ser637 and phosphorylated at Ser746 by p53/RIPK3 to induce alternative autophagy. Then Ulk1 induces the elongation of Golgi membranes and formation of isolation membranes under the activation of PI3K complex. Rab9 and Dram1 function essentially during the following closure of isolation membranes and generation of autophagosomes, which is similar as the role of Atg5, Atg7 and LC3 in canonical autophagy. Then autolysosomes form after the fusion of autophagosomes with lysosomes and then the cargoes are degraded

According to molecular mechanisms, autophagy is classified into canonical autophagy and alternative autophagy [8]. Since we focused on the mechanisms and roles of alternative autophagy, a comparison between canonical and alternative autophagy is briefly introduced here regarding to morphology, function, membrane source and substrates. Both types of autophagy show similar morphological and functional features on the whole. As described above, isolation membranes expand and the leading edges fuse to enclose components to be digested, which form autophagosomes [9]. Autophagosomes fuse with lysosomes to form autolysosomes and the components are degraded by lysosomal enzymes finally. An obvious difference is the membrane source, with canonical type originating from endoplasmic reticulum (ER) and mitochondria-associated ER membrane (MAM) [10] and alternative type from *trans*-Golgi membrane [2] (Fig. 1). A recent study from Torii et al. [11]identified the phosphorylation site of Unc-51-like kinase 1(Ulk1) at Ser746 is crucial for alternative autophagy, which means Ulk1 Ser746 phosphorylation induces alternative autophagy instead of canonical autophagy, despite Ulk1 is the initial regulator for both types. Also, different substrates are utilized by canonical and alternative autophagy. Specially, p62 is degraded by canonical autophagy. Secretory proteins such as insulin granules, and mitochondria within reticulocytes are degraded by alternative autophagy. Last but not the least, the participation of Atg5/Atg7 is an important characteristic for canonical autophagy, which differs from alternative autophagy. Hence, canonical autophagy is also called Atg5/Atg7 dependent autophagy and alternative autophagy is usually named as Atg5/Atg7 independent autophagy.

For a long time, Atg5/Atg7 were considered as essential for autophagy until Nishida et al [2] reported another type of autophagy, namely alternative autophagy, in wild-type mouse embryonic fibroblasts (MEFs) independent of Atg5. They found the biogenesis of autophagic structures in Atg5 knockout MEFs treated with etoposide, which were indistinguishable from the morphology in rapamycin-induced canonical autophagy. Autolysosomes were observed during etoposide- induced alternative autophagy by immunostaining with lysosome-associated membrane 2 (Lamp2). Bafiomycin A1 is an inhibitor of the fusion between autophagosomes and autolysosomes, and its addition clearly increased and decreased the formation of autophagosomes and autolysosomes respectively. The finding of alternative autophagy revealed a new regulatory mechanism and increasing attention are paying to the study of alternative autophagy and its role in different pathological processes (Table 1).

**Table1:** Studies on the role of alternative autophagy in different diseases

Animal / Cell	Stimulus/Chemical	Diseases/	Results	References
Ulk1-cKO mice Atg7-cKO mice Mito-Keima–Tg Parkin-KO mice CMs YFP-Rab9–Tg mice Rab9-KI mice	Starvation Glucose deprivation Hypoxia	Myocardial ischemia	① Mitophagy is induced by energy stress via an Atg7-independent butUlk1-dependent mechanism; ② Ulk1-dependent, but not Atg7-dependent mechanisms protect the heart against ischemic injury; ③ S179 phosphorylation of Rab9 plays an essential role in mediating the assembly of the Ulk1-Rab9-Rip1-Drp1 complex and activating mitophagy in the heart	[3]
DRPLA mice DN cells Human fibroblasts Neuroblastoma cells	Rapamycin Bafilomycin A1	DRPLA, one of the polyQ diseases	① Canonical autophagy is stalled in DRPLA mice and in human fibroblasts from patients of DRPLA; ② Alternative autopahgy is induced by chronic autophagy blockage in several conditions, including DRPLA and Vici syndrome; ③ The combination of alternative pathways and canonical autophagy blockade, results in dramatic nuclear pathology with disruption of the nuclear organization, bringing about terminal cell atrophy and degeneration	[53]
Ulk1^gt/gt^ mice Atg5 ^−^/^−^ mice Ulk1 ^gt/gt^ /Atg5^−^/^−^ mice Erythroblasts Reticulocytes Erythroid cells	3-Methyladenine Rapamycin Compound C Staurosporine	Erythrocyte differentiation; Stress erythropoiesis	① Alternative macroautophagy is responsible for mitochondrial clearance from embryonic reticulocytes,which is Ulk1- dependent and Atg5-independent; ② Ulk1-dependent alternative macroautophagy is also involved in stress erythropoiesis	[86]
TTFs MEFs pre-adipocytes iPSCs Atg5^−/−^ TTFs ULK1^−/−^ TTFs	Rapamycin AICAR SMER28 spermidine 3-Methyladenine Brefeldin A	iPSC reprogramming	① Robust iPSC reprogramming does not rely on canonical autophagy; ② Atg5-independent and Rab9/ULK1-dependent autophagy is required for reprogramming; ③ The Atg5-independent autophagy induced in reprogramming mediates mitochondrial clearance, by which metabolic switch towards glycolysis is facilitated	[88]
MEFs hMDMs J774	3-methyladenine Bafilomycin A Chloroquine Gentamicin	Francisella tularensis infection	① F. tularensis infection increases autophagic flux; ② Autophagy derived nutrients provide carbon and energy sources that support F. tularensis proliferation; ③ ATG5-independent macroautophagy may be beneficial to some cytoplasmic bacteria by supplying nutrients to support bacterial growth	[91]
colonic and intestinal epithelial cell lines Atg5^−/−^ or Atg7^−/−^ MEF cells	Starvation Chloroquine LPS MG132	Crohn’s disease; bacterial infection	① TRIM31 promotes LPS dependent autophagy in an Atg5- independent alternative process through directly interacting with PE in a palmitoylation-dependent manner; ② TRIM31 induces alternative autophagy, which is essential for eliminating intracellular pathogenic Shigella in intestinal cells; ③ Human cytomegalovirus-infected intestinal cells show a decrease in TRIM31 expression as well as a significant increase in bacterial load, reversible by the introduction of wild-type TRIM31	[6]

DRPLA, Dentatorubral-pallidoluysian atrophy; hMDMs, human monocyte derived macrophages; iPSCs, induced pluripotent stem cell; J774, J774A.1 macrophage-like cells; LPS,lipopolysaccharide; MEFs, Mouse embryonic fibroblasts; TRIM31, the tripartite motif 31; TTFs, Tail tip fibroblasts; PE, phosphatidylethanolamine;

Alternative autophagy could be induced by multiple Golgi stressors, such as etoposide [5], amphotericin B1 [12], genotoxic stress [2]. As mentioned above, the membranes of alternative autophagy originate from *trans*-Golgi apparatus and late endosomes [2]. The phosphorylation status of Ulk1 is quite important and it is phosphorylated by mammalian target of rapamycin complex 1 (at Ser637 and Ser757, mTOC1) and AMP-activated protein kinase (at Ser317, Ser467, Ser555, Ser777 and Thr574, AMPK) to keep inactivated under normal condition [13–15]. Upon stimulation of Golgi stress, Ulk1 is dephosphorylated at Ser637 and phosphorylated at Ser746 by p53/RIPK3 to induce alternative autophagy [11]. Then Ulk1 induces the elongation of Golgi membranes and formation of isolation membranes under the activation of PI3K complex. Rab9 [2] and Dram1 [16] function essentially during the following closure of isolation membranes and generation of autophagosomes, which is similar as the role of Atg5, Atg7 and LC3 in canonical autophagy [17]. Then autolysosomes form after the fusion of autophagosomes with lysosomes and then the cargoes are degraded. The exact mechanisms of alternative autophagy remain to be clarified.

## Difference and association between Canonical and alternative autophagy

The phosphorylation status of ULK1 is a key point for regulating both Canonical autophagy and alternative autophagy. ULK1 could form a complex together with Fip200, Atg13 and Atg 101. In physiological conditions, ULK1 is inactivated by phosphorylation of mTORC1 and AMPK at serine/threonine residues respectively[1, 7, 9]. When challenging with nutrient starvation, protein phosphatase 2A mediated ULK1 dephosphorylation resulted in translocation to pre-autophagosomal membranes for initiating the isolation membrane of Canonical autophagy. In the case of alternative autophagy, Mg^2+^/Mn^2+^ dependent 1D could mediate ULK1 dephosphorylation for triggering ULK1 complex formation together with Fip200, Atg13 and Atg 101 [1, 7, 9]. ULK1 complex further activates vesicle nucleation via promoting the class III PtdIns3K complex formation together with PtdIns3K, Beclin 1, Vps15 and Atg 14L [1, 7, 9]. Subsequently, Atg5/Atg7 mediates two ubiquitin-like conjugation system for driving elongation and closure of isolation membrane to form autophagosome [1, 7, 9]. In this process, Atg7 is indispensable for the conjugation of Atg12-Atg5 as an E1-like enzyme, and then Atg3 and Atg5-Atg12 acted as E2- and E3-like enzymes respectively for mediating phosphoatidylethanolamine conjugation to LC3 for transiation of LC3I to LC3II. According to these descriptions, it is not hard to conclude that Atg5/Atg7 is indispensable for formation of Canonical autophagosome [1, 7, 9]. Although both ULK1 and PI3K complexes are necessary for initiation of Canonical and alternative autophagy, Atg5/Atg7 have been demonstrated not to participate in alternative autophagy, and LC3-I to LC3-II conversion also doesn’t occur in alternative autophagy.

How were the elongation and closure of isolation membrane proceeded to form autophagosome in alternative autophagy without participation of Atg5/Atg7 associated two ubiquitin-like conjugation system? Autophagosome in alternative autophagy has been demonstrated to be associated with a Rab9-dependent manner, which could traffick proteins from late endosomes to *trans-*Golgi membranes to fuse isolation membranes with vesicles derived from the *trans*-Golgi and late endosomes [2]. In unstressed cells, Rab2 is primarily located on Golgi apparatus. After accepting autophagy stimuli, Rab2 could escape from Golgi apparatus and interact with ULK1 complex for facilitating the recruitment of ULK1 complex for autophagosome formation [18]. Group A *Streptococcus* (GAS) could inhibit canonical PIK3C3-dependent autophagy via secreting streptolysin O and Nga during early infection for promoting intracellular proliferation [19]. However, an alternative autophagy relying on RAB9A/RAB17-positive autophagosome formation could effectively engulf GAS and protest against infection [19]. Presumably, the members of GTPase including Rab9, Rab 2 and Rab17 are essential for extension and closure of isolation membranes in the alternative autophagy replacing the roles of Atg5/Atg7 in the Canonical autophagy. However, it remains to be further clarified in next experiments about how the members of GTPase are organized together and what the exactly mechanisms are for autophagosome formation in alternative autophagy.

After autophasome formation, UV radiation resistance associated gene (UVRAG) and the PtdIns3K facilitate autophagosome-lysosome fusion in Canonical autophagy. Syntaxin17 localizes to the outer membrane of matured autophagosomes and mediates autophagosome-lysosome fusion in the Canonical autophagy [20]. Because syntaxin 17 mainly localizes to completed autophagosomes not to isolation membrane [20], lysosome does not combine with the isolation membrane, which ensures the formation and degradation of autolysosomes [20]. However, the difference is that alternative autophagy presents that syntaxin7 not syntaxin17 has been demonstrated to colocalize to mannose-6-phosphate receptors (a trans-Golgi/late endosomal marker) and Lamp2 [2], which exhibits that syntaxin 7 might mediate autophagosome-lysosome fusion in the alternative autophagy. Besides, alternative autophagy doesn’t rely on UVRAG and PtdIns3K complex for facilitating autophagosome-lysosome fusion but depending on GTPase (Rab9) for autophagosome-lysosome fusion [2].

Both Canonical and alternative autophagy must form double-membraned vacuoles, called autophagosomes, to sequester substrates before delivering it to the lysosome for degradation [7, 9]. Canonical autophagy mainly targets and degrades most long-lived proteins, big protein complexes and intracellular organelles such as mitochondria. It also requires the participation of autophagy cargo receptor p62 (also termed SQSTM1), because p62 combines to ubiquitinated proteins via its ubiquitin-associated domain to LC3-II for promoting itself degradation in the process of Canonical autophagy [7, 9]. However, the current studies showed that alternative autophagy mainly phagocytosis and clear mitochondria in different conditions. Alternative autophagy could engulf and digest mitochondria in reticulocytes for promoting erythrocyte maturation. Enhancing alternative autophagy not Atg5-independent autophagy might promote induced pluripotent stem cells (iPSC) reprogramming via modulating mitochondrial clearance [21]. However, the Canonical and alternative autophagy are completely different from microautophagy and chaperone-mediated autophagy. The double-membraned autophagosome are not required in the latter two autophagy process. Micro-autophagy could directly deliver cellular constituents to lysosomes for degradation [22]. Micro-autophagy mainly maintains organellar size, membrane homeostasis and cell survival in conditions of nitrogen restriction [22]. Chaperone-mediated autophagy only recognizes soluble cytosolic proteins containing a KFERQ-like motif and deliver them to invagination in lysosome via a heat shock cognate 70 chaperone pathway [23].

## Roles of alternative autophagy in different diseases

Various diseases are caused by or related to abnormal accumulation of proteins and damaged organelles. Since autophagy exerts essential functions in degrading superfluous proteins and organelles, the role of autophagy in the physio-pathologic process of many diseases have been investigated. Until now, alternative autophagy has been emerging to be closely related to different diseases, such as cardiovascular diseases [3], neurodegenerative diseases [4], oncogenesis [5], and inflammatory bowel diseases [6] (Table 1).

### Mitochondria dysfunction and alternative mitophagy in heart diseases

Heart is an organ of high energy demand and mitochondria play a vital role in producing energy for maintaining cardiac functions. Normally, the numbers and functions of mitochondria maintain dynamic homeostasis by intrinsic mechanisms of biogenesis and mitophagy [24]. Mitochondrial dysfunctions have been proved to contribute to various heart diseases, especially ischemia reperfusion injury [25], diabetic cardiomyopathy (DCM) [26], pressure-overload induced cardiac hypertrophy and heart failure (HF) [27]. In the failing heart of experimental animals and humans, abnormal mitochondria were characterized as hyperplasia, fragmentation, electron-dense matrix loss and membrane disruption, accompanied with downregulation of mitofilin and cardiolipin [27]. In pressure overload induced hypertrophic hearts, mitochondria exhibit decreased numbers, increased fragmentation and distorted morphology characterized with blurred and ruptured membrane and cristae structures [28]. In diabetic cardiomyopathy, several mechanisms, including disordered mitochondrial Ca^2+^ uptake, reduced cardiac adiponectin actin, up-regulated O-GlcNAcylation, and dysregulated sirtuins’s activity, could contributes to mitochondrial dynamics and overexpression of reactive oxygen species [26]. Ischemia reperfusion injury could disproportionate mitochondrial fusion and fission, which impairs mitochondrial respiratory function and impacts mitochondria quality control [26, 29]. The resultant injuries of mitochondrial dysfunction are greatly related to insufficient ATP production and excessive ROS production, which conversely aggravate mitochondrial dysfunction through ETC damage, lipid peroxidation, and DNA break [30]. Thus, the autophagic clearance of damaged mitochondria is delicately performed by mitochondria specific autophagy, namely mitophagy, to decard impaired mitochondria [31]. Numerous studies have confirmed canonical mitophagy, which is dependent on Pink1/Parkin [32], exert important functions in cardiovascular diseases, such as myocardial infarction, ischemia–reperfusion and hypertrophy induced by pressure overload, heart failure and aging associated cardiomyopathy [33–36]. Althouhg Canonical mitophagy has been demonstrated to be one of the most important pathway for mitochondrial clearance, evidences have emerged to show that alternative autophagy that forms autophagosomes with trans-Golgi and late endosomes not the Canonical autophagy adapter LC3 have an extremely important role in mitochondrial clearance.

Study showed that LC3-II associated Canonical autophagy had been increased between 1 and 12 h after pressure overload induced by transverse aortic constriction (TAC), and then decreased below basal level 5 days after TAC surgery. However, electron microscopy observed that mitophagy was activated at 3–7 days post TAC coinciding with mitochondrial translocation of dynamin-related protein (Drp1) [37]. Based on this finding, it had been speculated that mitophagy might not dependent on Atg5/Atg7/LC3 mediated Canonical autophagy pathway. To discriminate whether mitophagy activation is dependent on Canonical autophay, wild type (WT) and Atg7-cardiomyocyte specific knockout mice (Atg7-cKO) mice were subjected to glucose starvation respectively [3]. As expected, LC3 mediated canonical autophagy flux was significantly increased in WT mice but was unable to be detected in Atg7-cKO mice. Despite the disappearance of LC3 associated flux in Atg7-cKO mice, electron micrographs remained to observe the mitophagy featured with double-membrane structures. Moreover, by relying on Atg7-cKO/Mito-Keima-Tg mice, authors once again confirmed that mitochondria were sequestered within autophagosomes in condition of starvation induced energy stress [3]. However, in the cardiomyocyte specific Ulk1 knockout mice (Ulk1-Cko), the LC3 protein mediated Canonical autophagy was intact, but mitochondrial clearance and mitophagy was completely inhibited [3]. These elegant experiments demonstrated that Ulk1-dependent alternative autophagy not Atg7/LC3 mediated anonical autophagy plays vital role in regulating mitophagy in energy stress conditions [3].

Nishida et al. have demonstrated that double membranes of autophagosomes derived from *trans*-Golgi and endosomes in a Rab9 dependent manner in alternative autophagy [2]. As expected, Saita T et al. demonstrated that Golgi-derived membranes inhibitor (brefeldin A) could significantly inhibit alternative autophagy in mouse heart and cardiomyocytes but showed none significant effect for canonical autophagy markers, and immunohistochemistry and Co-IP assays demonstrated that complex assembled by Ulk1, Rab9, Rip1and Drp1 has been in charge of recruiting *trans*-Golgi membranes to form mitophagy for mitochondria clearance and quality control [3]. Mechanistially, starvation or hypoxia/ischemia activates AMPK phosphorylation, which promote Ulk1 phosphorylation at serine 179. Phosphorylated Ulk1could further phosphorylate Rab9 at serine 179, which could promote Rip1 phosphorylation for forming complex of Ulk1, Rab9 and Rip1. This complex finally contributed to Drp1 phosphorylation resulted in mitophagy activation and cardioprotection during glucose deprivation and hypoxia/ischemia conditions [3]. Taken together, the protective mitophagy during energy stress in the heart was distinct from canonical autophagy and dependent on Ulk1-Rab9-Rip1-Drp1 regulated alternative autophagy. [3]

Rab9 belongs to RAS oncogene family and is involved in trafficking proteins and memberances [38]. Several members of this family have been showed to regulate mitochondria clearance through different pathways. Rab7 distributes on late endosomes, lysosomes, endoplasmic reticulum, *trans*-Golgi network and mitochondrial membranes [39, 40]. Activated Rab7 could direct autophagy related transmembrane protein ATG9a to damaged mitochondria for formatting autophagosome in Parkin-mediated mitophagy [39, 40]. Rab5-positive early endosomes could sequestrate Parkin-marked damaged mitochondria for delivering mitochondria to lysosomes for degradation [41]. Rab5-positive early endosomes mediated mitochondria clearance is started before canonical autophagy [41], which is different from the Rab9 mediated alternative mitophagy as described above, because Rab9-dependent alternative autophagy is Parkin independent but Rab5-dependent-alternative autophagy is Parkin dependent. Meanwhile, several proteins of Rab family have been confirmed to participate in the phagocytosis and killing of Group A Streptococcus (GAS) by alternative autophagy pathways. Rab9A and Rab23 are recruited into GAS containing autophagosme-like vacuoles (GcAVs), which is essential for the formation, maturation, and binding of GcAVs to lysosomes for degradation [42]. However, neither Rab9a nor Rab23 has been examined to localize to starvation-induced autophagosome formation [42]. This study demonstrated that Rab9A and Rab23 acted as alternative autophagy in response to GAS infection, which is different from starvation-induced canonical autophagy. Toh et al. have also found that GAS secreting Nga (NAD-glucohydrolase) could inhibit the PIK3C3-dependent conventioanl autophagy pathway for impairing the clearance of GAS, which could be compensated by the activation of alternative autophagy regulated by Rab9/Rab17A to kill GAS [19]. Taken together, studies imply the potential roles of Rab family in regulating alternative autophagy during physiopathological process in different context.

### Alternative autophagy in neurodegenerative diseases

Neurodegenerative diseases, including Parkinson disease (PD), Alzheimer’s disease (AD), amyotrophic lateral sclerosis (ALS) and polyQ, are characterized by the aggregation of misfolded proteins resulted in neurons injuries [43]. Autophagy is the most important intracellular process for degrading misfolded proteins and damaged organelles in neurodegenerative diseases [4]. Therefore, autophagy has been recognized as one of the most important mechanisms in regulating diseases severity. Investigators have presented that both canonical and alternative autophagy have been involved in the pathology of neurodegenerative diseases [4, 44].

Atg7-dependent canonical autophagy is necessary for the survival of midbrain dopaminergic (mDA) neurons in the substantia nigra pars compacta (SNpc). Deletion of Atg7-dependent canonical autophagy contributed to reduction of mDA neurons resulted in progress of Parkinson’s disease (PD) [45] In 1-Methyl-4-phenyl-1,2,3,6-tetrahydropyridine (MPTP) induced neurotoxin mouse models, deletion of Atg7-dependent canonical autophagy is associated with accumulation of p62 and ubiquitin marked proteins [45]. That is to say, Atg7 regulated canonical autophagy was needed for mDA neurons survival in physiological condition and was also one of the most important mechanisms for degrading unfolded proteins to protest against MPTP-induced mDA neuron degeneration. The canonical autophagy does not only degrade the unfolded proteins but also cleans up damaged mitochondria in PD [46]. Studies have demonstrated mitochondrial damage is associated with the progress of PD, however, PINK1 could recognize and accumulate on the damaged mitochondria for activating and recruiting Parkin to the outer membrane of damaged mitochondria for mitochondrial clearance resulted in attenuating PD disease [46]. For the pathogenesis of AD, abnormal production of amyloid-beta (Aβ) and hyperphosphorylated tau protein are hallmarks [47]. Researchers are focusing on revealing autophagy related strategies to clear Aβ and tau in recent years but controversial results were obtained [48, 49]. Inducing autophagy to enhance the degradation of Aβ showed beneficial effects in some studies but others found autophagy is involved in the development of AD and its inhibition would decrease the secretion of Aβ [48–50].

The canonical autophagy is significantly decreased in a mouse neuron degenerative model of polyglutamine disease dentatorubral-pallidoluysian atrophy (DRPLA) evidenced by accumulation of P62 and up-regulation of LC3-I/II ratio [51]. The inhibition of canonical autophagy induced activation of alternative autophagy pathway including Golgi membrane-associated autophagy and nucleophagy [51]. Niemann-Pick type C disease (NPC) is a neurodegeneration disease characterized with an accumulation of cholesterol and glycosphingolipids in late endosomes and lysosomes [52]. Mouse model of NPC disease exhibited that canonical autophagy has been significantly increased evidenced by increased lapidated LC3 and accumulated autophagosomes, however, the autophagosome-lysosome fusion seemed to be impaired resulted in decreased substrate degradation. Therefore, the disproportion of autophagsomes induction and flux formation in the canonical autophagy contributed to autophagic stress resulted in neuronal dysfunction and cell death in NPC disease [52, 53]. However, Rab9 overexpression in NPC cells could effectively reduce endosomal/lysosomal accumulation of cholesterol and sphingolipids [54]. This implied that Rab9-dependent alternative autophagy might be very important for attenuating NPC disease. The mechanisms of alternative autophagy in the pathology of NPC still need further investigation and activating alternative autophagy is considered to be a promising therapy in NPC.

AD is a progressive degeneration neuropathy characteristed with deterioration of mental activities and eventually a vegetative state [55]. Several causative factors have been demonstrated to involve in the development and progress of AD, including accumulation of amyloid-β (Aβ), tau protein and AβPP, loss of neurons and alteration of the cytoskeleton [55]. The Golgi apparatus presented a morphological and morphometric alteration in twenty AD brains. It is well established that Golgi play a vital role in protein trafficking and misfolding protein degradation in an alternative autophagy dependent pathway [55]. Tau hyperphosphorylation is one of the most remarkable features in AD. Study has indicated that neuronal Golgi fragmentation appears before the occurrence of tau hyperphosphorylation [56], moreover, the Golgi fragmentation was significantly increased in an age dependent manner and was positively correlation with tau hyperphosphorylation in mouse brains [56]. Overexpression of Golgi matrix protein (golgin-84) could effectively attenuate Golgi fragment resulted in decreased tau hyperphosphorylation and accumulation [56]. Phosphorylation of Golgi stacking protein GRASP65 lead to dysfunction of Golgi structure formation resulted in exaggerated Golgi fragmentation. Inhibiting GRASP65 phosphorylation could prevent Aβ-accumulation induced Golgi fragment and reduce Aβ production resulted in improved AD. These studies exhibited that disorder of Golgi structure and function in neurons might directly impair traficking, degrading and sorting of a variety of proteins. The Golgi apparatus fragment was also examined in motor neurons of patients with amyotrophic lateral sclerosis (ALS) and in mice models of ALS [57]. Exaggerated Golgi apparatus fragment also exhibited an exacerbated ALS, therefore, the fragment of apparatus might be associated with the neuronal degeneration in ALS [57]. It is well established that Golgi play a vital role in protein trafficking and misfolding protein degradation in an alternative autophagy dependent pathway. Thus, Golgi apparatus associated alternative pathway might play important role in regulating proteolysis of protein systems in AD. The interaction of Golgi defects and pathogenesis of neurodegenerative diseases is quite intricate. Whether alternative autophagy is a protective mechanism or the pathological change of diseases after the initiate of Golgi disruption remain to be elucidated.

As mentioned above, etoposide associated genetoxic stress could induce autophagy in Atg5−/− MEFs. Surprisingly, etoposide treatment could no longer induce formation of autophagosomes and autolysosomes in Atg5−/− Wipi3−/− MEFs [58]. This confirmed that Wipi3 is an essential protein for inducing Atg5-independent alternative autophagy. Wipi3 was dispersed in the cytoplasm in control MEFs, whereas Wipi3 could translocate to the *trans*-Golgi after etoposide treatment [58]. Wipi3-mediated alternative autophagy mainly eliminates proteins that accumulate in condition of genotoxic stress. Neuron-specific Wipi3-conditional deficient mice (Wipi3^cKO^) showed lower motor performance than wild type mice [58]. Neuropathological analyses presented that Wipi3^cKO^ suffered from severe cerebellar and cerebral cortex degeneration characterized with increased glial fibrillary acidic protein (GFAP), accumulated lamellar bodies and enhanced dead neuron cells. Although the decreased alternative autophagy in Wipi3^cKO^, the canonical autophagy markers of Atg7 and p62 showed none significant difference between Wipi3^cKO^ and its littermate. Despite the similar neurological defects in Wipi3^cKO^ and Atg7-neuron-specific-knockout (Atg7^cKO^) mice [59, 60], the organellar morphologies showed great difference. Small fragment and swollen rod-shaped Golgi membranes could be seen frequently in purkinje cells of Wipi3^cKO^ mice, whereas the Golgi apparatus in Atg7^cKO^ showed intact. However, the purkinje cells in Atg7^cKO^ were scattered a plenty of ER membranes, which also indicated the inhibition of Atg7-medated canonical autophagy. Finally, authors exhibited that Wipi3 deletion lead to accumulation of abnormal mitochondria and exaggerated ferroptosis in neuron cells [58], thus Wipi3-induced alternative autophagy mainly eliminate abnormal mitochondria and inhibit ferroptosis in genetoxic stress-induced n eurodegenerative disease.

### Alternative autophagy in oncogenesis

p53 is a well-known tumor suppressor gene. p53 is the most frequently altered gene in many cancer types, including ovarian cancers, breast cancers, oesophageal cancers, small-cell lung cancers and squamous cell lung cancers [61]. As an important transcription factor, p53 is responsible for regulating the expression of genes related to proliferation, DNA repair, senescence and cell death [62, 63]. p53 also interacts with autophagy via multiple pathways in the context of tumor. Autophagy suppressed p53 by reducing oxidative stress and preventing DNA damage, which is important for promoting tumor. In a mouse model of lung cancer, tumor cells with Atg7 deficiency showed p53 activation, increased cell death and decreased tumor burden compared with cells with intact Atg7 [64]. Similar evidence was obtained after observing mouse model for non-small cell lung cancer after Atg7 deletion, namely the progression to adenocarcinomas was altered to benign oncocytomas with autophagy deletion induced-p53 activation [65]. However, the function of p53 on autophagy could be activated or inhibited. Firstly, p53 could activate autophagy via transcriptional and non-transcriptional mechanisms [66]. Several autophagy -relating genes have been proved to be modulated directly by p53, including Dram, Isg20L1, Ulk1 and Atg7 [67–69]. p53 could also activate autophagy through regulating AMPK-mTOR pathway [70]. Besides, p53 has been demonstrated to play important role in regulating alternative autophagy.

Etoposide-induced genotoxic stress caused decreased Ulk1 phosphorylation at Ser637 in p53 intact (p53+/+) MEFs, however, Ulk1 phosphorylation of Ser637 showed none significant different in etoposide-treated p53 knockout (p53−/−) MEFs compared to that in p53+/+ MEFs [10]. Because Ulk1 phosphorylation is a common initial step of canonical and alternative autophagy, it is crucial to investigate how p53 could mediate Ulk1 phosphorylation in condition of genotoxic stress. PPM1D had been a potential candidate to mediate Ulk1 phosphorylation, because it has been demonstrated that PPM1D could be activated in a p53-dependent pathway in conditions of genotoxic stress [71]. As expected, PPM1D expression was significantly up-regulated in p53+/+  MEFs rather than in p53−/− MEFs after etoposide treatment. In the same instant with the upregulation of PPM1D and downregulation of Ulk1 phosphorylation in etoposide treated cells, the autophagy maker LC3-II was significantly increased accompanied with markedly decreased p62 protein [10]. However, etoposide treatment could no longer induce autophagy after PPM1D deletion (PPM1D−/−) in MEFs. Interestingly, starvation associated energy stress could not induce the change of PPM1D expression, meantime the LC3-II expression and autophagic flux showed none any difference between PPM1D+/+ MEFs and PPM1D−/− MEFs. This implied that genotoxic stress induced an alternative autophagy pathway which might be different from the starvation-induced canonical autophagy. The PPM1D/ULK1 mediated alternative autophagy mainly depressed irradiation-induced thyocyte apoptosis via degradating the proapoptotic Noxa protein. Surprisingly, this alternative autophagy could not degrade Noxa protein in thymocytes lacking Atg7 [10]. This implied that this genotoxic stress induced alternative autophagy was also different from Atg7-independent alternative autophagy.

However, another study indicated that etoposide treatment could induced the generation of autolysosomes in Atg5−/− MEFs [11]. The generation of autolysosomes could not be examined in the Atg5−/− Ulk1−/− MEFs. Moreover, the phosphorylated Ulk1 at Ser746 was co-localized with Golgi marker GS28 [11]. These experiments were reasonable to deduce that etoposide treatment induced an atg5-independent alternative autophagy after Ulk1 phosphorylation at Ser746. The target sequence of Ulk1 at Ser^746^ possessed a similar sequence with RIPK3 [72]. Immunoprecipitation demonstrated that RIPK3 and Ulk1 combined together in untreated MEFs, etoposide treatment could significantly increase the combination between RIPK3 and Ulk1. RIPK3 deletion could inhibit etoposide-induced alternative autophagy accompanied with reduction of Ulk1 phosphorylation at Ser746. Thus, RIPK3 mediated Ulk1 phosphorylation is necessary for genotoxic stress-induced generation of alternative autophagy. As presented above, etoposide treatment induced Ulk1 dephosphorylation in a p53/PPM1D dependent manner. The authors also demonstrated that p53/PPM1D mediated Ulk1 dephosphorylation at Ser637 was also essential for Ulk1 Ser746 phosphorylation induced alternative autophagy [11]. Furthermore, etoposide treatment could promote RIPK3 upregulation in Atg5−/− MEFs, however, RIPK3 expression was significantly down-regulated in Atg5−/− MEFs consistent with p53 deletion. The Ulk1 phosphorylation at Ser746 was vanished in p53−/− MEFs treated with etoposide. So, genotoxic stress induced RIPK3 activation was dependent on p53 regulation. Collectively, p53 played a dual role in the genotoxic stress induced alterative autophagy via phosphorylating Ulk1 at ser 637 relying on p53-PPM1D axis but dephosphorylating Ulk1 at ser746 depending on p53-RIPK3 axis. It has been figured out that genotoxic stress could induce Golgi morphology alteration resulted in disrupted Golgi trafficking [12] also presented that P53/Ulk1 mediated alternative autophagy might take part in eliminating superfluous undelivered proteins in etoposide treated MEFs. However, the roles of p53/Ulk1 mediated alternative autophagy remains to be investigated in future studies.

### Alternative regulated inflammatory bowel disease

Inflammatory bowel disease (IBD), including Crohn’s disease (CD) and ulcerative colitis (UC), is characterized with abnormal inflammatory and immune responses in the intestine [73, 74]. Genome-wide associated investigations indicated that numerous canonical autophagy genes have involve in IBD [75, 76]. Previous studies have been mainly focused on canonical autophagy and have been working to develop IBD treatment based on restoring canonical autophagy [75], because canonical autophagy defects have shown to disrupt intestinal homeostasis, affect gut microbiota composition, hamper intracellular bacterial clearance and increase intestinal inflammation [75]. Recently, TRIM31 (tripartite motif) mediated alternative autophagy has been revealed to involve in protecting against pathogenic bacterial infection in IBD [6]. TRIM31 has been suggested to specially localize with the mitochondria and lysosomes in intestine. Remarkably, TRIM31 was decreased by 45% in patients with Crohn’s disease compared healthy people [6]. TRIM31 enriched in mitochondria and decreased in IBD lead authors to investigate whether TRIM31 took part in regulating autophagy [6]. Unexpectedly, TRIM31-positive autophagosomes occurred in Atg5−/− cells and were further increased in Atg5−/− cells treated with LPS stimulation [6]. Further study showed that TRIM31-mediated autophagosomes/autolysosomes formation didn’t need participation of Atg7, Beclin1, and LC3 [6]. As a matter of fact, TRIM31 directly combined to phosphatidylethanolamine (PE) in a palmitoylation-dependent manner for promoting autophagic membrane association and autolysosomal formation [6]. These experiments implied that TRIM31-mediated alternative autophagy might compensate for the loss of Atg5/Atg7 associated canonical autophagy. Subsequently, authors found alternative autophagy induced by TRIM31 was capable of restricting the growth of Shigella in epithelial cells and eliminating intracellular Shigella in intestinal cells. Meanwhile, cytomegalovirus (HCMV) infection significantly downregulated the expression of TRIM31 and thus inhibited the clearance of intracellular Shigella, which may explain the reason for positive correlation of IBD severity with HCMV infection [6]. Thus, TRIM31-mediated alternative autophagy could effectively protect against pathogenic bacterial invasion of intestine for improving IBD.

### Alternative autophagy regulate cell differentiation

In addition to heart diseases, neurodegeration, oncogenesis, alternative autophagy also participated in regulating cell differentiation by eliminating mitochondria in embryonic reticulocytes and induced pluripotent stem cells (iPSCs) [77]. As early as 2008, Mondira Kundu et al [80] identified Ulk1 as the critical regulator of mitochondrial clearance during the final stages of erythroid maturation, however, overexpression of Ulk1 could not induce autophagy in condition of nutrient deprivation. They suggested that alternative machinery could have led to the elimination of mitochondria in erythroid cells rather than atg5/atg7 dependent canonical autophagy. Mitochondria clearance is the final stage of erythrocyte maturation. Autophagy has been demonstrated to eliminate mitochondria from reticulocytes, because altra-structural observation has confirmed that autophagic vacuoles engulfed the mitochondria in reticulocytes [77]. Interestingly, autophagic vacuoles remained to engulf and eliminate mitochondria in reticulocytes after deletion canonical autophagy protein Atg5, however, none autophagic vacuoles could be observed for engulfing and eliminating mitochondria in reticulocytes in Ulk1-deficent and Ulk1/Atg5 double-deficient mice [77]. This definitely showed that mitochondria elimination in reticulocytes was not dependent on Atg5 regulated canonical autophagy but Ulk1 dependent alternative autophagy pathway [77]. Rab9 and syntaxin7 (makers for alternative autophagy) rather than LC3 were co-localized with Lamp2 in reprogramming induced pluripotent stem cells (iPSCs) [21]. Knockdown fo ULK1 or Rab9 using shRNAs could significantly inhibit the generation of iPSCs, however, Atg5 knockdown showed none significant inhibition of iPSCs generation [21]. This suggested that Rab9/ULK1 dependent alternative autophagy rather than Atg5-dependent canonical autophagy was involved in regulating the generation of iPSCs. Successful generation of iPSCs needs metabolic switch from mitochondrial oxidative phosphorylation to glycolysis in the reprogramming process [21]. ULK1/Rab9 dependent alternative autophagy mediated mitochondrial clearance could effectively elimit mitochondria resulted in facilitating the metabolic switch from mitochondrial to glycolysis [21].

### Alternative autophagy regulated *Francisella tularensis* proliferation

*Francisella tularensis* (*F. tularensis*) is a facultative intracellular bacteria that could easily infect more than 200 different species including humans. *F. tularensis* could replicate up to 100-fold within 24 h after infecting host cells, thus the mortality would be 30–60% in pneumonic patients infected with *F. tularensis* without effective treatment [78]. In cultured primary macrophages, *F. tularensis* promote the formation of autophagosome-like structure with multi-membranous, so it has been suggested that autophagy might be essential for the rapid replication in host cells [79]. To examined whether autophagy has provided the rapid replication of *F. tularensis,* three autophagy inhibitors including 3-methyladenine (3MA), BA and CQ could significantly reduce *F. tularensis* growth in MEF, however, amino acid supplementation could restore bacterial growth in autophagy inhibitors treated cells [78]. This raised the interesting possibility that autophagy supplied a source of amino acid for supporting *F. tularensis* rapid replication. Examining mTOR/S6 kinase activity was used to evaluate the induction of canonical autophagy. Although the mTOR/S6 kinase activity was significantly inhibited after 8 h of infection, the bacterial replication had significantly increased at 3 h afte *F. tularensis* infecting host cells [78]. Thus , the time of mTOR/S6 kinase inhibition-mediated autophagy was inconsistent with the inition of *F. tularensis* rapid replication. Moreover, *F. tularensis* growth could not be impaired in Atg5−/− MEFs compared to none Atg5 deletion MEFs [78]. As a matter of fact, the *F. tularensis* replication in Atg5−/− MEFs seemed to be markedly increased [78]. Taken together, these experiments showed that rapid replication of *F. tularensis* was supported by an Atg5-independent altenative autophagy. Atg5-independent alternative autophagy is sentive to *trans*-Golgi apparatus inhibitor Brefeldin A (Bref A), because the membrances of alternative autophagy autophagosomes derived from the *trans*-Golgi apparatus. *F. tularensis* replication was significantly inhibited in cells treated with Bref A. This once again confirmed the alternative autophagy rather than Atg5 dependent canonical autophagy have supported the rapid replication of *F. tularensis* in infected host cells. Thus, accurate inhibition of alternative might be a potential stagey for protesting against *F. tularensis* infection.

## Conclusions and future directions

From above description, we could clearly know that alternative autophagy has been demonstrated to involve in different disease and biology process. The alternative autophagy seemed to be induced only in conditions of cellular stress rather than basal conditions. However, the formation mechanisms and biological roles of alternative autophagy are far from being elucidated. There are at least following questions about alternative autophagy demand prompt solution in the future studies.

The roles of alternative autophagy seemed to play a key role in eliminating mitochondria in ischemia insulted cardiomyocytes, erythrocytes mature and differentiate and iPSCs induction. This implied that alternative autophagy might be a highly selective autophagy rather than a consist autophagy as canonical autophagy acting. This raises several issues for future investigation and consideration. Firstly, what are the mechanisms that direct alternative autophagy rather than canonical LC3/Parkin-dependent autophagy for eliminating mitochondria? Secondly, does the canonical autophagy and alternative autophagy coexist or has the alternative autophagy been activated only for compensating the canonical autophagy after inhibition or block of canonical autophagy in specific conditions? Thirdly, Drp1 has been demonstrated to be a major pro-fission protein for regulating damaged mitochondria clearance [81]. How does the alternative autophagy discriminate the dysfunction mitochondria for eliminating? Does the alternative autophagy also recognize the Drp1 mediated mitochondria fission as the canonical autophagy acting?

PtdIns 3-Kinase combined with Becin1 promotes the initiation of canonical autophagy, however, quantitative analyses using a cross-linker showed that about 50% of PtdIns3-kinase is free from Beclin1 interaction [82]. Immunofluorescence microscopy showed that majority of PtdIns 3-kinase localize to the *trans*-Golgi or the late endosome which have been suggested to promote the formation of alternative autophagy [82]. Without the Beclin1 participation, what molecular have replaced the role of Beclin1 in promoting initial formation of autophagy complex by combining with PtdIns 3-Kinase? Although the difference of upstream regulation pathway and the membrane composition between alternative and canonical autophagy, the serine/threonine kinase Ulk1 has been suggested to be indispensable in the initial step of both conditions. The different phosphorylation sites in Ulk1 have showed to dominate the initiation of canonical or alternative autophagy. AMPK/mTORC1 signaling-mediated Ulk1 phosphorylation at Ser637 contributes to the initiation of starvation associated canonical autophagy [83], however, genotoxic stress induced Ulk1 phosphorylation at Ser746 and dephosphorylation at Ser637 has directed the generation of *trans*-Golgi associated alternative autophagy [11]. It is reasonable to deduce that regulation of multiple phosphorylation sites in Ulk1 might lead to generation of different alternative autophagy. It has been reported that more than 70 phosphorylation sites have been identified in Ulk1 [11]. Are additional kinases waiting to be investigated in regulating Ulk1 phosphorylation resulted in some other more alternative autophagy pathways?

Although the roles of alternative autophagy have been suggested to involve in protecting against ischemia associated mouse heart injuries, promoting erythrocytes mature and inhibited pathogenic bacterial invasion of intestine for improving IBD, the roles and mechanisms of alternative autophagy in different pathophysiological states is far from being elucidated. For example, Ulk1/Rab9 mediated alternative autophagy could prevent mouse hearts from ischemia injuries. Is Ulk1/Rab9 associated autophagy able to regulate alternative mitophagy in other pathological conditions such as pressure overload induced cardiac hypertrophy, doxorubicin-induced myocardial remodeling, diabetic cardiomyopathy, and heat failure? Tumor suppressor p53 has been demonstrated to involve in varieties of cellular processes, including DNA repair, cell-cycle regulation, cellular senescence, apoptosis and autophagy [84]. P53 had been demonstrated to play a dual role in process of canonical autophagy associated cancer development [84]. This review presented that p53 regulated alternative autophagy might have been emerging in tumors but its roles in tumors’ development and progress remained to be established. Besides, whether does the p53 associated alternative autophagy play different regulatory roles in different tumors? Uncovering the underlying mechanisms of 53 between canonical and alternative autophagy might possess important implications for treating various tumors.

Alternative autophagy has also been demonstrated to involve in regulating bacterial phagocytic and cellular differentiation and mature. Canonical autophagy has been demonstrated to be essential in each step of dendritic cells maturation, including antigen presentation, migration and cytokine secretion [85]. Does alternative autophagy could play similar roles in regulation of immune cells mature? Some invasive pathogens have developed smart strategies to escape xenophagy and LC3-phagocytosis [85]. GAS secreting Nga inhibits canonical autophagy mediated degradation, however, Rab9/Rab17A-medated alternative autophagy could compensate the role of the missed canonical autophagy for degrading GAS infection. It will be a very important issue to investigate whether alternative autophagy could replace and compensate the roles of xenophagy and LC3-phagocytosis for degrading invasive pathogens.

As discussed above, research about alternative autophagy has just been launched. Further elucidation of the mechanisms and biological roles of alternative autophagy will require more well designed experiments. One of the key points is that we must exclude the roles of canonical autophagy firstly before exploring the roles and mechanisms of alternative autophagy. Besides, it also be very important that we should elucidate the correlation between canonical autophagy and alternative autophagy. Therefore, a deeper understanding of the roles and mechanisms of alternative autophagy should depend on knockout mice with targeted deletion of genes specific to canonical or alternative autophagic pathway in future studies.

## Data Availability

Not applicable.

## Abbreviations

ER: Endoplasmic reticulum
Ulk1: Unc-51-like kinase 1
Lamp2: Lysosome-associated membrane 2
HF: Heart failure
TAC: Transverse aortic constriction
GAS: Group A Streptococcus
Nga: NAD-glucohydrolase
AD: Alzheimer’s disease
mDA: Midbrain dopaminergic
Aβ: Amyloid-beta
NPC: Niemann-Pick disease type C
PICALM: Phosphatidylinositol-binding clathrin assembly protein
ZMC1: Zinc metallochaperone-1
iPSC: Induced pluripotent stem cells
IBD: Inflammatory bowel disease
PE: Phosphatidylethanolamine
MAM: Mitochondria associated endoplasmic reticulum membrane
MEFs: Mouse embryonic fibroblasts
DCM: Diabetic cardiomyopathy
PGC-1α: Peroxisome proliferator-activated receptor coactivator-1α
CMs: Cardiomyocytes
GcAVs: GAS containing autophagosme-like vacuoles
PD: Parkinson disease
ALS: Amyotrophic lateral sclerosis
SNpc: Substantia nigra pars compacta
DRPLA: Dentatorubral-pallidoluysian atrophy
APP: Amyloid precursor protein
PRIMA-1: P53 reactivation and induction of massive apoptosis-1
PPM1D: Protein phosphatase magnesium-dependent 1D
Bref A: Brefeldin A
TRIM: Tripartite motif
HCMV: Cytomegalovirus
